# Mapping of Brain Activity in the Analgesia Induced by Ph*α*1*β* and Morphine

**DOI:** 10.3389/fmolb.2021.770471

**Published:** 2022-02-04

**Authors:** Danuza Montijo Diniz, Carlos Malamut, Marina Rios Araújo, Andrea Vidal Ferreira, Juliana Figueira Silva, Marta do Nascimento Cordeiro, Marcia Helena Borges, Marco Aurélio Romano Silva, Marcus Vinicius Gomez, Célio Jose Castro Junior

**Affiliations:** ^1^ Department of Neurotransmitters, Santa Casa, Institute of Education and Research, Belo Horizonte, Brazil; ^2^ Radiobiology Department, Center for the Development of Nuclear Technology, National Commission of Nuclear Energy (CDTN/CNEN), Belo Horizonte, Brazil; ^3^ Department of Biochemistry, Ezequiel Dias Foundation, Belo Horizonte, Brazil; ^4^ Department of Mental Health, Faculty of Medicine, Federal University of Minas Gerais, Belo Horizonte, Brazil

**Keywords:** P. E. T, pain, morphine, Ph*α*1*β*, brain, 18F-FDG

## Abstract

Preclinical evidence suggests the potential of Ph*α*1*β*, a toxin obtained from the venom of spider *Phoneutria nigriventer*, as a new analgesic drug. Molecular brain imaging techniques have afforded exciting opportunities to examine brain processes in clinical pain conditions. This paper aims to study the brain regions involved in the analgesic effects of Ph*α*1*β* compared with Morphine, in a model of acute pain induced by formalin in Sprague Dawley rats. We used ^18^F-fluorodeoxyglucose as a metabolic radiotracer to perform brain imaging of rats pretreated with Ph*α*1*β* or Morphine in a model of acute inflammatory pain caused by intraplantar injection of formalin. The rats’ hind paw’s formalin stimulation resulted in a brain metabolic increase at the bilateral motor cortex, visual cortex, somatosensory cortex, thalamus, and cingulate cortex.In rats treated with Ph*α*1*β*, selective inhibition of unilateral motor cortex and cingulate cortex was observed. Morphine treatment leads to small and selective inhibition at the bilateral amygdala striatum and accumbens. Our results indicate that the analgesic effect of Ph*α*1*β* and Morphine possesses a differential profile of central processing in the pain state.

## Introduction

Ph*α*1*β* has a broad impact on inhibiting high-voltage activated calcium channels (HVCCs) (Vieira et al., 2005) and as an antagonist of the transient receptor potential cation channel, TRPA1 (Tonello et al., 2017). Phα1β presents antinociceptive efficacy in several rodent pain models (Souza et al., 2008; Souza et al., 2008; de Souza et al., 2011; Castro-Junior et al., 2013; Rigo et al., 2013a; Rigo et al., 2013b; de Souza et al., 2014; Diniz et al., 2014; Rosa et al., 2014; Tonello et al., 2017). Considerable research in the last decade has focused on N-type calcium channel inhibitors to develop novel analgesic drugs. As it has been shown in preclinical trials, Ph*α*1*β* has a wider therapeutic window than ω-conotoxin MVIIA, ziconotide, Prialt® for the pain treatment (Souza et al., 2008; de Souza et al., 2011) and has the potential to become a new analgesic drug. The better analgesic profile of Phα1β compared with ω-conotoxin MVIIA can be explained by several factors, including different affinities of the toxin for the activated states of N-type VSCCs and binding in other types of calcium channels (Lewis et al., 2000; Vieira et al., 2005; Winquist et al., 2005; Altier et al., 2007). Therefore, Ph*α*1*β* can block pain with more efficacy due to its ability to interact with multiple targets of calcium channels in nociceptive pathways (Vieira et al., 2005). Despite the involvement of Phα1β in the control of HVCCs of sensory afferent neurons (Castro-Junior et al., 2013) or the spinal cord (Souza et al., 2008), nothing is known about how this peripheral inhibition of neurotransmission by Ph*α*1*β* can control the activation of other superior areas of the central nervous system (C.N.S.).

The drug development process is a lengthy, high risk and costly endeavor. Although each step’s specificity and duration depend on the target indication and the drug class, in general, clinical development and investigation of a new drug submitted to regulatory approval take no less than 10 years. Molecular imaging approaches can be used in the initial stages of drug development in clinical trials such as positron-emission tomography, P.E.T. Most large pharmaceutic companies have now established molecular imaging as an integral part of both research and development. There are many expectations that molecular imaging technology investments will enhance drug development (Rudin, 2005). This technique fulfils an essential criterion for a translational approach to drugs acting on pain. Molecular imaging can allow the non-invasive assessment of biological and biochemical processes in living subjects. Such technology, therefore, has the potential to enhance our understanding of analgesic drug activity during preclinical and clinical drug development, which could aid decisions to select analgesic candidates that seem most likely to be successful.

Therefore, we performed a comparative study with ^18^FDG, a P.E.T. radiopharmaceutical, to investigate brain glucose metabolism changes during the development of pain induced by formalin and its effect on analgesic drugs. This paper aims to study the brain regions involved in the analgesic effects of Ph*α*1*β* in comparison with Morphine in a model of acute pain induced by formalin in rats.

## Materials and Methods

### Animals

Thirty-four adult male Wistar rats 8–10 weeks old; weighing approximately 250–280 g) were used in the present study. Rats were housed in a well-controlled environment with a 12 h light/dark cycle and constant humidity and temperature. They were housed in plastic cages at three animals per cage with free access to food and water. The experiments were performed following the current guidelines for the care of laboratory animals and the ethical guidelines for investigations of experimental pain in conscious animals (Zimmermann, 1983). Also, following the National Institutes of Health guide for the care and use of Laboratory animals (N.I.H. Publications No. 8023, revised 1978). All efforts were made to minimize animal suffering and to reduce the number of animals used. The Ethics Committee of the Federal University of Minas Gerais authorized the studies (Protocol 347/2012).

### Drugs

Na_2_HPO_4_, KH_2_PO_4_ and NaCl used to prepare PBS and 37% formaldehyde to prepare the formalin. Reagents were purchased from Sigma Chemical Co., (St. Louis, United States.A). Morphine sulfate and isoflurane anaesthetic, from Cristália laboratory (São Paulo, Brazil).

**FIGURE 1:**
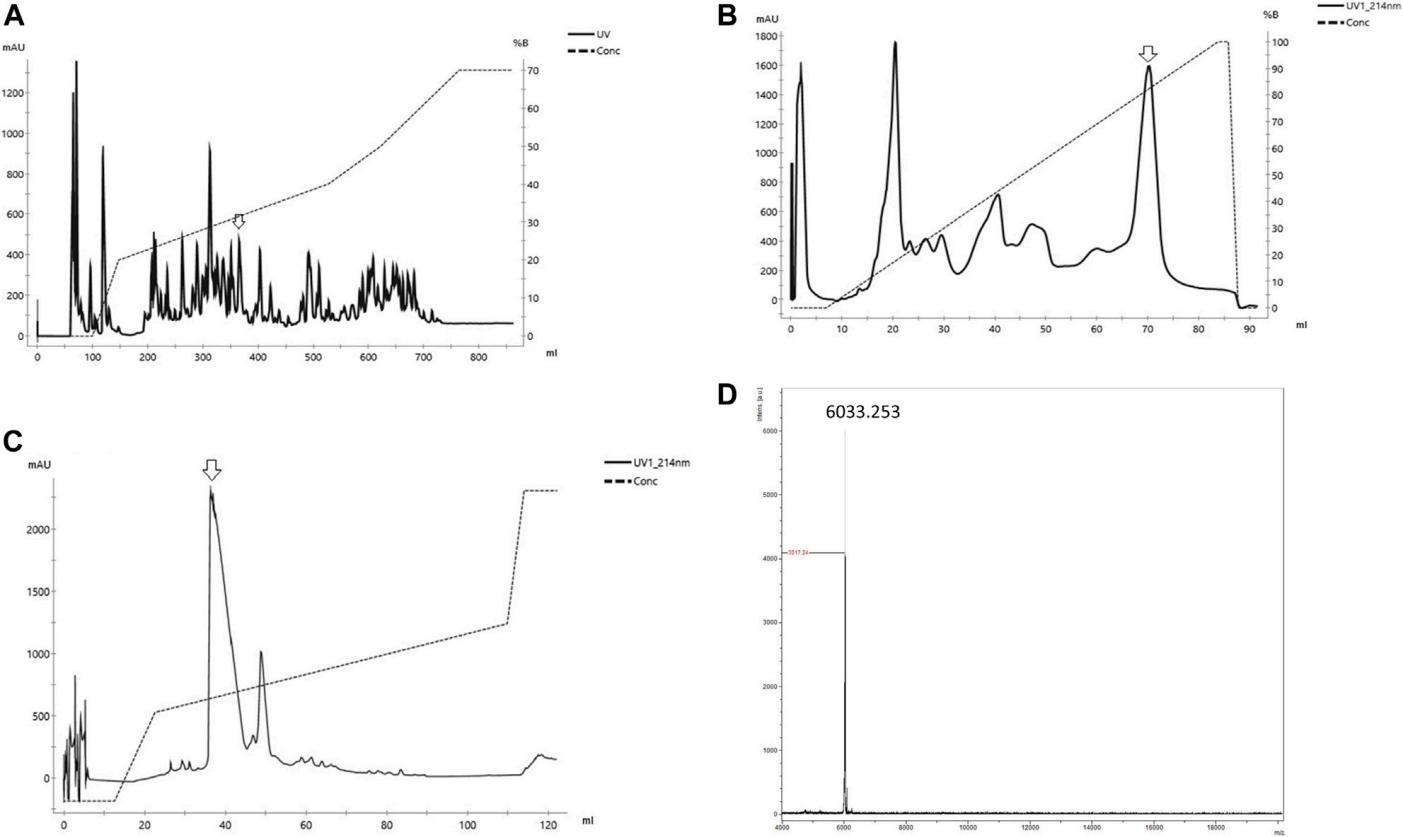
Purification of Ph*αβ* from the venom of the spider *P. nigriventer* **(A)** Reverse phase of the crude venom of *Phoneutria nigriventer* spider in column C4 Vydac. **(B)** Cation exchange HPLC of the fraction Phαβ from **(A)** on Hi trap Sp Hp column **(C)** Reverse phase desalting of Phαβ from **(B)** in a column C18 small pore (arrow indicates the peak used in each step) **(D)** Mass spectra of the Phαβ MS obtained on MALDI/TOF in positive/linear mode (laser frequency 100%, mass range 2–20,000 (*m/*z).

Ph*α*1*β* was purified from spider venom (Figure 1) according to the technique described by (Cordeiro et al., 1993). *P. nigriventer* venom was obtained by electrical stimulation of anesthetized spiders. Venom was centrifuged at 4000*g* for 10 min, and the supernatant was fractionated by gel filtration on columns of Sephadex G-50 superfine and superose 12HR, and reverse phase fast protein liquid chromatography on C2/C8 (PEP-RPC) and C1/C8 (PRO-RPC) columns as described in detail previously (Cordeiro et al., 1993). Peptides were detected by monitoring the absorbance at 216 nm (Cordeiro et al., 1993). The fraction PhTx3 obtained from the chromatography on a PRO-RPC column was dissolved in 1 ml of 0.1% (v/v) aqueous trifluoroacetic acid (TFA) and subjected to reverse phase HPLC on a preparative column (22 mm _ 25 cm) of Vydac C18 (218TP1022; Technicol Ltd, Stockport, UK) equilibrated in the same solvent. The column was eluted with a linear gradient (0–40% over 180 min) of acetonitrile (HPLC grade S; Rathburn Chemical Co., Peebles, Scotland, UK) in 0.1% TFA at a flow rate of 10 ml min_1. We collected three fractions (A–C; Cordeiro et al., 1993), and fraction C was dissolved in 1 ml of 10 mM sodium phosphate buffer, pH 6.5, and fractionated on a weak cation exchange HPLC column (4.6 mm _ 25 cm) of Synchropak CM 300 (Synchrom Inc, Lafayette, IN) equilibrated in the same buffer. After absorption, the columns were eluted with a linear gradient (0–0.5 M NaCl over 90 min) in the same buffer at a flow rate of 2 ml min_1. The toxin Ph*α*1*β* eluted at a salt concentration of 0.16 M (Figure 1), and was desalted by absorption onto Sep-Pak C18 cartridges (Waters, Milford, MA), which were then washed with 15 ml of 0.1% TFA, and Ph*α*1*β* was eluted with 5 ml of acetonitrile containing 0.1% TFA.

### Intrathecal Injections

According to the previously described method, the intrathecal injections within the spinal cord levels of L5 and L6 (Hylden and Wilcox, 1980). Briefly, using a 28-G needle connected to a 10 µL Hamilton microsyringe to administer medication (5 µL/site, i. t) lightly restrained the animal from maintaining the needle’s position. A slight tail-flick indicated the puncture of the dura. All experiments were performed in a single-blind manner to avoid possible observer bias results.

### Imaging Experiments

The images to obtain brain maps of formalin-induced nociception in the rats by P.E.T. ^18^F-FDG was used as a radiotracer to allow visualization of glucose metabolism in the brains. The ^18^F-FDG (70 MBq, averaged activity) was administered intraperitoneally (i.p). After that, the rats returned to boxes in a quiet environment to recover from i. p. injection and subsequent uptake of ^18^F-FDG. The fixed time for ^18^F-FDG uptake was 1 h. Initially, the rats received 50 µL of formalin (5%) or PBS into the right hind paw 30 min after ^18^F-FDG injection. This method was performed according to procedures described by (Dubuisson and Dennis, 1977). An initial set of experiments was performed (PBS or formalin group) to obtain the map of formalin-induced nociception in the rat brain. On another set of experiments, the effect of the drugs treatments was tested. The rats received intrathecal (i.t) administration of Phα1β (100 pmol/site), morphine (10 μg/site), or vehicle (PBS, 10 μL/site, control group) 1 hour before ^18^F-FDG injection. Therefore, the time between drug treatment and formalin injection was 1:30 h, which was enough time for the drugs to reach antinociceptive efficacy. After 55 min of ^18^F-FDG infusion, the animals were lightly anaesthetized with a mixture of isoflurane and oxygen and fixed their body in the microPET equipment (LabPET4, GE) coupled to an anaesthesia system to maintain anaesthesia during imaging acquisition. The rats’ body temperature (35.5°C) was maintained with a custom-made heating bed warmed with perfused hot water. Images obtained in the MicroPET for 15 min total, three head-bed positions (5 min/each).

### Image Processing

To establish a relation between the number of counts per second per voxel (cps/voxel) and the activity in Bq/cm3, after the acquisition of animal’s images, an image of a phantom with known activity, weight and volume, was performed at every day of the experiment and corrected the radioactive decay of ^18^F-FDG automatically during the procedures. The processing and reconstruction of images for the evaluation of ^18^F-FDG uptake in the brain were done by selecting volumes of interest (V.O.I.) using the PMOD II software (PMOD Technologies, Adliswil, Switzerland). Analysis of rat brain data on selected brain regions performed by normalizing entry data with the V.O.I. template using Px Rat (W.Schiffler) atlas (Schiffer et al., 2006). Data were expressed as S.U.V. (standard uptake value). The images decay-corrected measurement of the MBq/ml radiotracer uptake obtained *via* P. E. T. They were normalized by the amount of activity injected and the mass in kilograms of the animal being studied. When appropriate, S. U. V. values were later normalized by the % control group intake, as indicated in the text.

### Statistical Analysis

The quantification based on the S. U. V. values of each V. O. I. obtained. The importance of S. U. V.'s of each region represents the average of the five pixels with greater intensity. When indicated, the S. U. V. 's of each V. O. I. were normalized by the percentage of the control group’s capture (formalin in the paw and pretreated with intrathecal PBS or drug) and stratified by the different brain structures of each mouse. To evaluate if the data present a normal distribution, the Kolmogorov-Smirnof test was used. Repeated-measures ANOVA was used to examine whether formalin stimulation induced metabolic changes in the corresponding brain regions in both hemispheres, with the significance level set at *p<*0.05. Factorial ANOVAs were used to assess differences in 18F-FDG uptake among the groups with formalin stimulation pre treated with PBS, formalin stimulation with Ph*α*1*β* pretreatment, and formalin stimulation with morphine pretreatment, with *p*<0.05 again considered to be significant. Fisher’s post hoc tests were used to assess differences between groups.

## Results

### Brain ^18^F-FDG Uptake Associated With Formalin Nociceptive Stimulus

**FIGURE 2:**
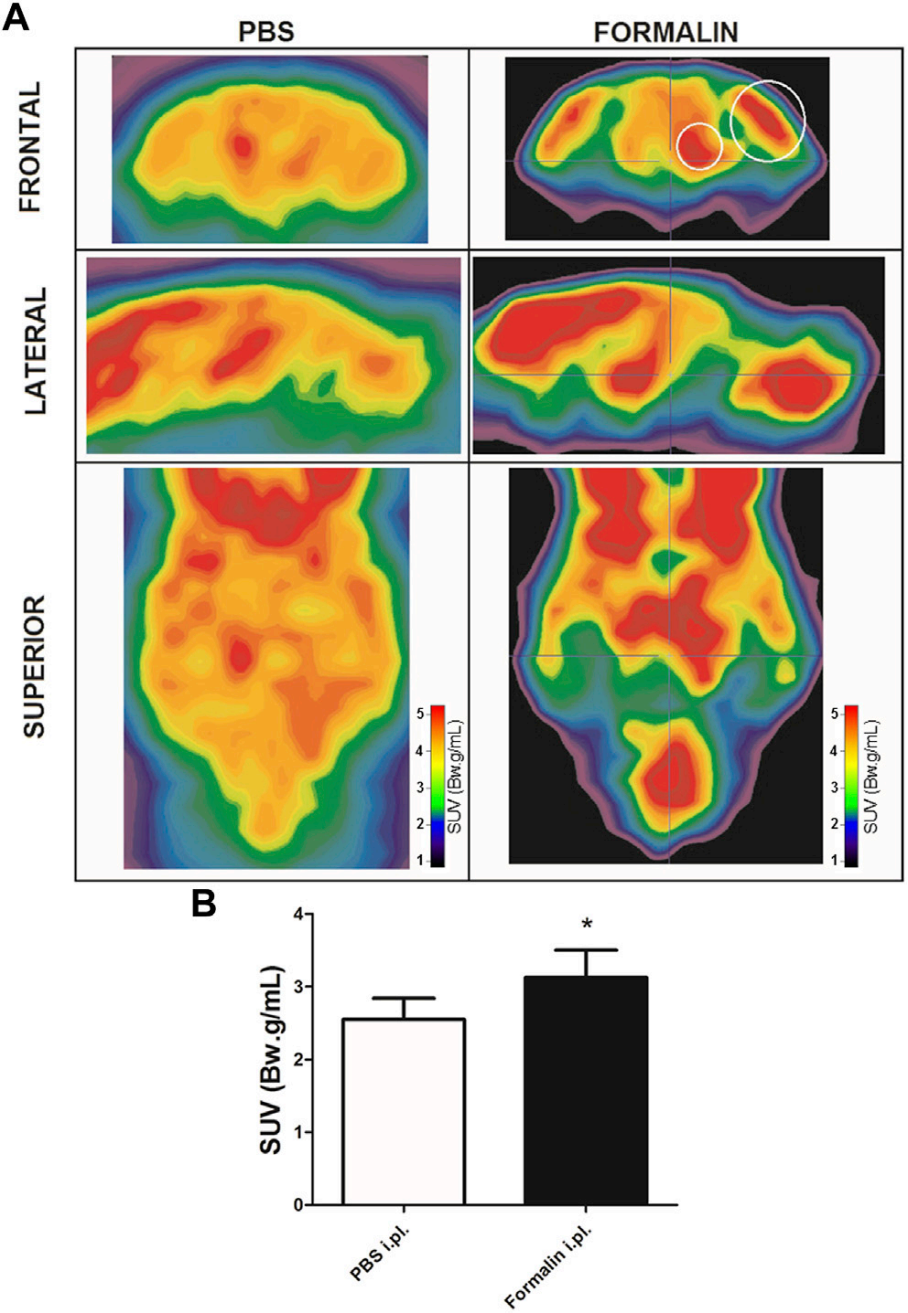
Formalin-induced cerebral metabolic activation. **(A)** Representative microPET images of brains from rats injected with PBS (left column) or with 5% Formalin (right column) into the right hind paw. Images are presented in different planes, as indicated. Warmer colors indicate higher uptake of ^18^F-FDG, hence higher metabolic activity. Small and large white circles depict regions that contains thalamus and somatosensory cortex, respectively. **(B)** averaged ^18^F-FDG uptake of the whole brain in rats that received PBS (white bar, N = 4) or 5% Formalin (black bar, N = 6) into the right hind paw. Bars represent mean+S.E.M. of the S.U.V. values (*p = 0.05, t-student test).

**FIGURE 3:**
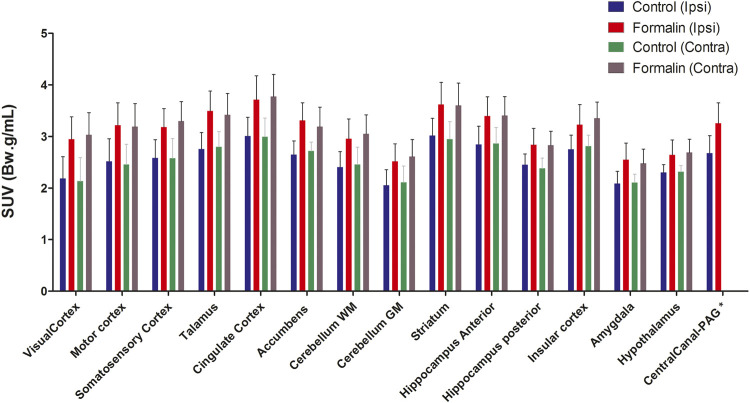
Differential uptake of ^18^F-FDG in brain areas associated to formalin-induced nociceptive stimulus. The different brain areas are presented bellow *x* axis. The definition of contra or ipsilateral was made in reference to the right paw where formalin or PBS was administered. Bars represent mean+S.E.M. of the S.U.V. values (N = 4 and N = 6, PBS and formalin groups, respectively) (N = 4 and N = 6, PBS and formalin groups, respectively. Repeated-measures ANOVAs with Fisher’s post hoc tests). WM, white mater; GM Gy mater; PAG periaqueductal gray.

Intraplantar injection of formalin (5%) into the right hind paw induced greater 18F-FDG brain uptake (Figure 2). The S.U.V. values for the whole brain were 2.55 ± 0.6 and 3.13 ± 0.8 Bw. g/mL in the groups that received PBS or intraplantar formalin, respectively (*p* = 0.05, t-student test). No significant differences were observed comparing the whole left and whole right hemisphere in the brain of animals that received formalin (data not shown). Posterior analysis revealed that different brain regions presented different uptake intensities (Figure 3 and Table 1). Thirty-three regions were analysed, 25 of them divided according to the cerebral hemisphere (ipsi or contralateral concerning formalin injection), and eight of them are central (under a sagittal plane) were not divided into ipsi or contralateral. The most significant differences in formalin-induced uptake were observed in the following regions: visual cortex, motor cortex, somatosensory cortex, thalamus, and cingulate cortex. Less expressive differences in formalin-induced ^18^F-FDG uptake were observed in the pituitary, medulla, hypothalamus, pons, and prefrontal cortex. No significant laterality differences were observed in formalin-induced ^18^F-FDG uptake in any of the areas studied (Figure 3 and Table 1). Although it was observed a pattern of higher ^18^F-FDG uptake induced by formalin in all brain regions ranging from approximatelly 10–20%, no significant difference (*p* > 0.05) was seen for any of the regions.

**TABLE 1:** ^18^F-FDG uptake in brain regions: Mean values of S. U. V.+S. E. M. Of the groups receiving PBS or formalin (i.pl.), in the two brain hemispheres.

Region	Ipsilateral		Contralateral	
	Control (PBS)	Formalin	Control (PBS)	Formalin
AcbCore/Shell	2.65 ^+^ 0.27	3.30 ^+^ 0.35	2.72 ^+^ 0.18	3.19 ^+^ 0.38
Amygdala	2.09 ^+^ 0.24	2.55 ^+^ 0.33	2.11 ^+^ 0.17	2.48 ^+^ 0.28
CaudatePutamen	3.01 ^+^ 0.34	3.62 ^+^ 0.43	2.95 ^+^ 0.34	3.60 ^+^ 0.43
CortexAuditory	2.50 ^+^ 0.25	3.00 ^+^ 0.34	2.47 ^+^ 0.23	3.12 ^+^ 0.36
CortexCingulate	3.01 ^+^ 0.36	3.71 ^+^ 0.47	3.00 ^+^ 0.37	3.78 ^+^ 0.43
CortexEntorhinal	2.40 ^+^ 0.29	2.91 ^+^ 0.39	2.39 ^+^ 0.20	2.88 ^+^ 0.37
CortexFrontalAssociation^a^	2.35 ^+^ 0.33	2.96 ^+^ 0.47		
CortexFrontal^a^	2.43 ^+^ 0.45	3.09 ^+^ 0.44		
CortexInsular	2.75 ^+^ 0.27	3.22 ^+^ 0.40	2.81 ^+^ 0.21	3.35 ^+^ 0.39
CortexMedialPrefrontal	3.06 ^+^ 0.21	3.61 ^+^ 0.35	3.06 ^+^ 0.22	3.53 ^+^ 0.36
CortexMotor	2.52 ^+^ 0.44	3.22 ^+^ 0.44	2.45 ^+^ 0.40	3.19 ^+^ 0.45
CortexOrbitofrontal	3.02 ^+^ 0.37	3.64 ^+^ 0.50	2.93 ^+^ 0.31	3.53 ^+^ 0.042
CortexParA	2.28 ^+^ 0.43	2.98 ^+^ 0.42	2.24 ^+^ 0.48	3.11 ^+^ 0.44
CortexRetrosplenial	2.39 ^+^ 0.43	3.20 ^+^ 0.47	2.31 ^+^ 0.43	3.17 ^+^ 0.46
CortexSomatosensory	2.58 ^+^ 0.36	3.18 ^+^ 0.36	2.58 ^+^ 0.39	3.30 ^+^ 0.38
CortexVisual	2.18 ^+^ 0.43	2.95 ^+^ 0.43	2.13 ^+^ 0.46	3.03 ^+^ 0.43
HippocampusAnteroDorsal	2.84 ^+^ 0.35	3.40 ^+^ 0.37	2.86 ^+^ 0.31	3.40 ^+^ 0.37
HippocampusPosterior	2.45 ^+^ 0.21	2.84 ^+^ 0.32	2.38 ^+^ 0.20	2.83 ^+^ 0.27
Hypothalamus	2.30 ^+^ 0.15	2.64 ^+^ 0.29	2.31 ^+^ 0.12	2.70 ^+^ 0.25
Olfactory	2.69 ^+^ 0.28	3.33 ^+^ 0.45	2.69 ^+^ 0.21	3.27 ^+^ 0.43
SuperiorColliculus	2.80 ^+^ 0.40	3.40 ^+^ 0.40	2.78 ^+^ 0.33	3.39 ^+^ 0.32
Midbrain	2.59 ^+^ 0.25	3.19 ^+^ 0.37	2.67 ^+^ 0.19	3.19 ^+^ 0.34
VTA	2.30 ^+^ 0.14	2.71 ^+^ 0.29	2.25 ^+^ 0.10	2.74 ^+^ 0.27
CB-grey	2.06 ^+^ 0.30	2.51 ^+^ 0.34	2.11 ^+^ 0.32	2.61 ^+^ 0.33
CB-white	2.40 ^+^ 0.31	2.95 ^+^ 0.39	2.45 ^+^ 0.34	3.05 ^+^ 0.37
InferiorColliculus	2.82 ^+^ 0.34	3.46 ^+^ 0.35	2.69 ^+^ 0.29	3.41 ^+^ 0.35
ThalamusWhole	2.75 ^+^ 0.32	3.50 ^+^ 0.38	2.80 ^+^ 0.30	3.42 ^+^ 0.42
Pituitary^a^	1.92 ^+^ 0.13	2.12 ^+^ 0.26		
CB-bloodflow^a^	3.07 ^+^ 0.38	3.70 ^+^ 0.51		
CentralCanal-PAG^a^	2.68 ^+^ 0.34	3.25 ^+^ 0.40		
Pons^a^	2.18 ^+^ 0.11	2.51 ^+^ 0.26		
Septum^a^	2.59 ^+^ 0.37	3.27 ^+^ 0.46		
Medulla^a^	2.31 ^+^ 0.13	2.59 ^+^ 0.24		

a Regions located in a central plane between the two cerebral hemispheres. They are not categorised as right or left by the analysis software, although they are presented as ipsilateral in the table. The definition of contra or ipsilateral was made in reference to the right paw where formalin or PBS, was administered.

### Effect of Ph*α*1*β* on the Uptake of ^18^F-FDG in Animals Injected With Formalin

**FIGURE 4:**
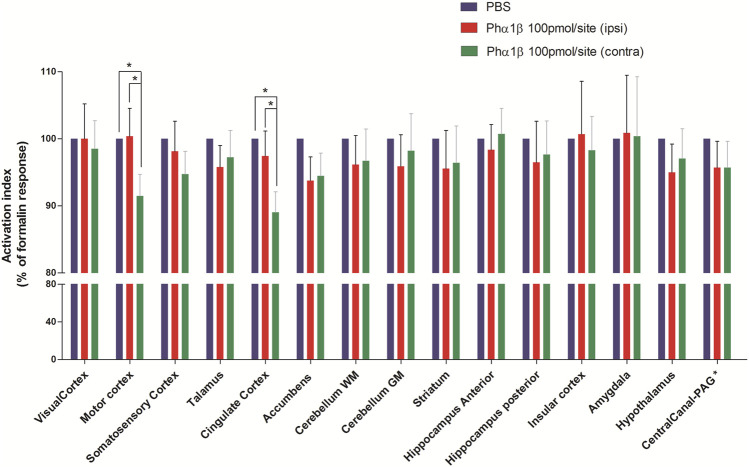
Effect of pretreatment with Phα1β toxin on metabolic changes in different brain areas of rats induced by formalin. Bars represent mean+S.E.M. ^18^F-FDG uptake normalised by the ^18^F-FDG uptake in the group that received intrathecal PBS. Pretreatment with intrathecal Ph*α*1*β* (100 pmol/site) led to a reduction in the metabolic activity in some neuroanatomic regions, notably in the cingulate cortex and motor cortex. The definition of contra or ipsilateral was made in reference to the right paw where formalin or PBS was administered (N = 6 and N = 10; PBS and Ph*α*1*β*, respectivelly). * denotes *p* < 0.05, Factorial ANOVAs with Fisher’s post hoc tests). WM: white matter, GM: gray matter, PAG: periaqueductal gray.

In another set of experiments, animals were injected with Ph*α*1*β* toxin (100 pmol/site, intrathecal) 1 h and 30 min before induction of nociception by formalin and then subjected to P.E.T. imaging. The increase in ^18^F-FDG uptake induced by formalin was normalised as to 100% in every brain areas with the 0% being ^18^F-FDG uptake in animals pretreated with intrathecal PBS rather than Phα1β or Morphine. This formalin-induced activation of the contralateral cingulate cortex and contralateral motor cortex regions were most significantly inhibited by Phα1β (89 ± 3% and 91 ± 3.2% of the control, respectively, *p* < 0.05 compared to the respective ipsilateral region) (Figure 4 and Table 2). Other areas such as accumbens, somatosensory cortex, thalamus, hypothalamus and striatum showed inhibitions in the uptake of ^18^F-FDG compared with control animals treated with PBS, but no lateralization of the inhibitory effect was seen for these regions.

**TABLE 2:** Effect of treatment with Ph*α*1*β* or Morphine on formalin-induced brain activation: comparison between brain regions according to the cerebral hemisphere. Values represent mean ± S. E. M. of^18^F-FDG uptake normalised by uptake in the intrathecal PBS group.

Region	Ipsilateral		Contralateral	
	Ph*α*1*β*	Morphine	Ph*α*1*β*	Morphine
AcbCore/Shell	93.78 ^+^ 3.52	94.46 ^+^ 3.32	94.47 ^+^ 3.40	91.18 ^+^ 3.16
Amygdala	100.87 ^+^ 8.63	93.22 ^+^ 5.60	100.39 ^+^ 8.88	89.11 ^+^ 4.59
CaudatePutamen	95.56 ^+^ 5.67	92.77 ^+^ 3.11	96.43 ^+^ 5.49	94.32 ^+^ 3.51
CortexAuditory	102.03 ^+^ 5.14	98.01 ^+^ 4.74	98.51 ^+^ 4.29	96.16 ^+^ 4.16
CortexCingulate	97.42 ^+^ 3.74	96.73 ^+^ 4.05	89.04 ^+^ 3.04	91.16 ^+^ 4.58
CortexEntorhinal	95.16 ^+^ 4.69	90.73 ^+^ 4.86	99.55 ^+^ 4.69	89.46 ^+^ 4.60
CortexFrontalAssociation^a^	98.87 ^+^ 6.36	98.20 ^+^ 6.21		
CortexFrontal^a^	101.43 ^+^ 4.23	99.61 ^+^ 5.05		
CortexInsular	100.70 ^+^ 7.86	94.36 ^+^ 3.51	98.31 ^+^ 5.02	94.36 ^+^ 3.51
CortexMedialPrefrontal	96.40 ^+^ 3.57	97.60 ^+^ 5.23	94.27 ^+^ 4.26	97.64 ^+^ 6.09
CortexMotor	100.39 ^+^ 4.15	96.91 ^+^ 2.99	91.49 ^+^ 3.19	92.07 ^+^ 3.98
CortexOrbitofrontal	98.62 ^+^ 5.59	97.39 ^+^ 3.21	100.79 ^+^ 3.71	98.90 ^+^ 3.39
CortexParA	100.71 ^+^ 510	99.16 ^+^ 4.89	98.72 ^+^ 4.57	101.93 ^+^ 4.14
CortexRetrosplenial	98.42 ^+^ 5.05	97.55 ^+^ 5.10	96.89 ^+^ 4.94	96.25 ^+^ 3.98
CortexSomatosensory	98.13 ^+^ 4.50	94.74 ^+^ 3.96	94.73 ^+^ 3.38	94.50 ^+^ 4.51
CortexVisual	100.01 ^+^ 5.18	100.63 ^+^ 4.86	98.52 ^+^ 4.17	100.60 ^+^ 3.96
HippocampusAnteroDorsal	98.35 ^+^ 3.79	98.23 ^+^ 4.20	100.72 ^+^ 3.81	101.34 ^+^ 3.87
HippocampusPosterior	96.48 ^+^ 6.13	96.83 ^+^ 5.47	97.66 ^+^ 5.01	94.61 ^+^ 3.00
Hypothalamus	95.01 ^+^ 4.20	95.38 ^+^ 4.18	97.06 ^+^ 4.44	95.29 ^+^ 5.31
Olfactory	93.92 ^+^ 5.09	90.52 ^+^ 4.72	98.94 ^+^ 4.44	93.69 ^+^ 2.76
SuperiorColliculus	96.63 ^+^ 4.23	97.53 ^+^ 4.88	96.48 ^+^ 4.14	98.49 ^+^ 4.16
Midbrain	99.29 ^+^ 3.92	98.42 ^+^ 4.33	99.66 ^+^ 5.70	98.19 ^+^ 4.49
VTA	97.03 ^+^ 4.89	104.24 ^+^ 6.14	95.44 ^+^ 4.60	97.71 ^+^ 5.66
CB-grey	95.91 ^+^ 4.70	101.00 ^+^ 5.40	98.23 ^+^ 5.53	103.29 ^+^ 6.00
CB-white	96.14 ^+^ 4.34	100.97 ^+^ 4.32	96.74 ^+^ 4.71	100.57 ^+^ 5.23
InferiorColliculus	97.53 ^+^ 5.04	96.22 ^+^ 3.90	96.10 ^+^ 3.63	99.96 ^+^ 5.33
ThalamusWhole	95.79 ^+^ 3.22	96.28 ^+^ 4.38	97.26 ^+^ 3.97	96.28 ^+^ 4.38
Pituitary^a^	98.44 ^+^ 6.32	97.90 ^+^ 9.98		
CB-bloodflow^a^	98.52 ^+^ 4.93	98.78 ^+^ 4.34		
CentralCanal-PAG^a^	95.70 ^+^ 3.90	97.40 ^+^ 4.95		
Pons^a^	97.3 ^+^ 5.28	100.92 ^+^ 5.93		
Septum^a^	97.32 ^+^ 4.24	95.54 ^+^ 3.84		
Medulla^a^	97.86 ^+^ 6.43	99.87 ^+^ 4.94		

a Regions located in a central plane between the two cerebral hemispheres. They are not categorised as right or left by the analysis software, although they are presented as ipsilateral in the table. The definition of contra or ipsilateral was made in reference to the right paw where formalin or PBS, was administered.

### Effect of Morphine on the Uptake of ^18^F-FDG in Animals Injected With Formalin

**FIGURE 5:**
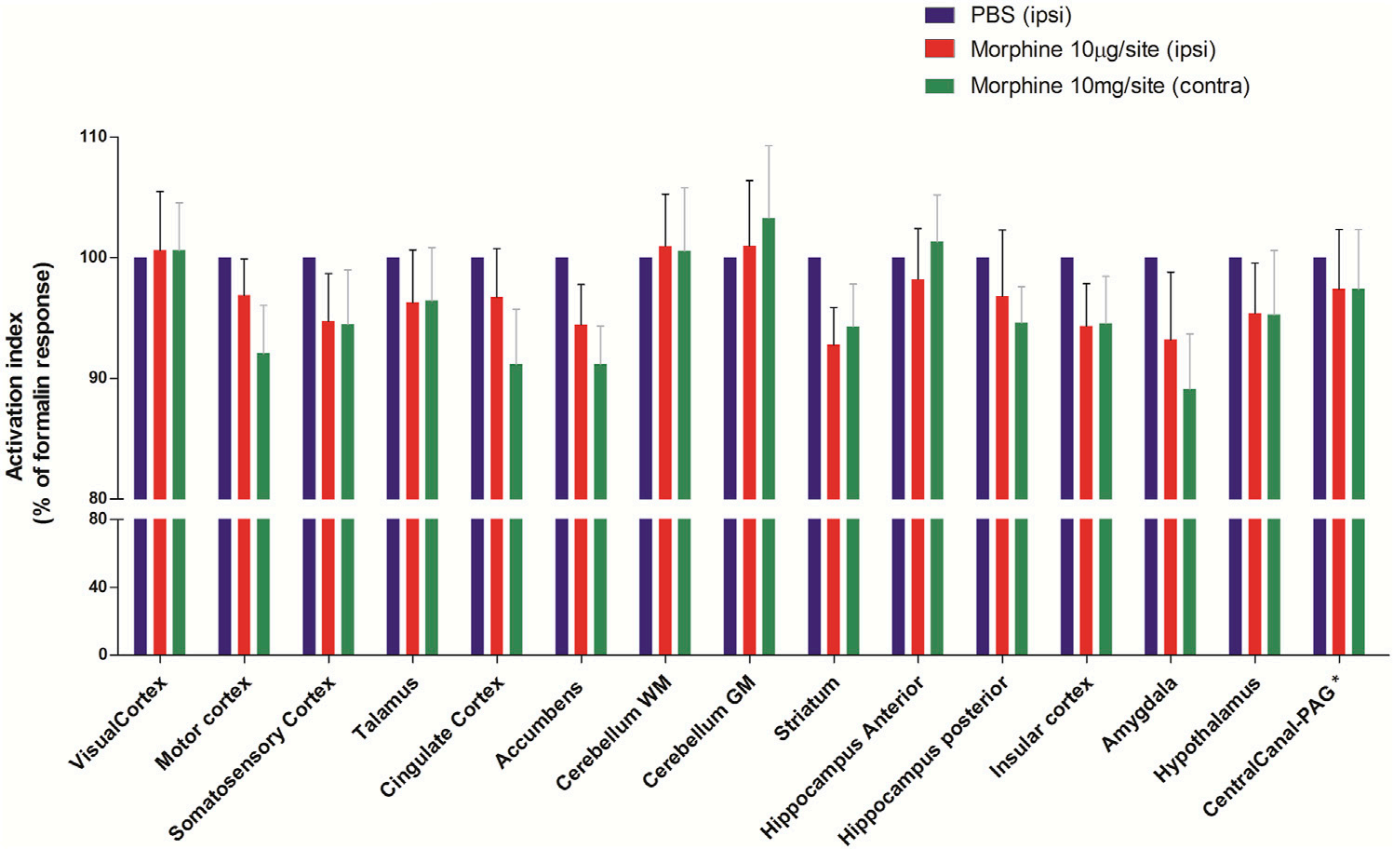
Effect of pretreatment with Morphine on metabolic changes in different brain areas of rats induced by formalin. Bars represent mean+S.E.M. ^18^F-FDG uptake normalised by the ^18^F-FDG uptake in the group that received intrathecal PBS. Pretreatment with intrathecal Morphine (10 µg/site) led to a reduction in the metabolic activity in some neuroanatomic regions, notably in the amygdala, striatum and accumebens. The definition of contra or ipsilateral was made in reference to the right paw where formalin or PBS was administered (N = 6 and N = 8, PBS and Morphine groups, respectivelly. ANOVAs with Fisher’s post hoc tests). WM: white matter, GM: gray matter, PAG: periaqueductal gray.

Morphine (10 μg/site, intrathecal) was injected 1.5 h before the induction of nociception by formalin and then submitted to P. E. T. imaging. Contralateral cingulate cortex, contralateral accumbens and contralateral amygdala were most significantly inhibited by Morphine (91+4.6%, and 91+3.2% and 89+4.6% of the control, respectively, *p* > 0.05 compared to the respective ipsilateral region) (Figure 5 and Table 2). Other regions such as the somatosensory cortex, thalamus, hypothalamus, insular cortex and striatum showed inhibition of uptake with the control-treated with PBS. Pretreatment with Morphine did not cause lateralization of the inhibitory effect at any of the analyzed areas.

## Discussion

This work’s data reveal a prominent and bilateral activation of several brain regions induced by the acute pain process, indicating these regions’ participation in the central processing of pain. More expressive metabolic activations were observed in the visual cortex, motor cortex, somatosensory cortex, cingulate cortex, thalamus and nucleus accumbens. Both the Ph*α*1*β* toxin and Morphine, via intrathecal injection, attenuated the pain-induced metabolic activation in these areas. However, inhibition by Phα1β occurred mainly in the anterior cingulate cortex and motor cortex’s contralateral hemisphere. Morphine was also able to attenuate metabolic activity in these areas, mainly in the amygdala and nucleus accumbens.

Nociceptive information is transmitted from the spinal cord to the brain through different pathways. Consequently, various regions of the brain are activated during the complex experience of pain. Several imaging studies in both humans and rodents examine cortical and subcortical areas involved in pain processing. Although there are many differences in activation patterns among the studies, there is a consistent activation pattern that includes sensory, limbic and associative areas (Bushnell et al., 1999). Based on previous studies using either P.E.T. or fMRI techniques, the most commonly associated regions are primary and secondary somatosensory cortex, anterior cingulate cortex, insular cortex, prefrontal cortex, thalamus, and cerebellum.

Brain areas with differential activations in pain depending on whether these areas receive direct or indirect nociceptive inputs. For example, the primary and secondary somatosensory cortex receives nociceptive and non-nociceptive information from the thalamus (Friedman and Murray, 1986; Shi and Apkarian, 1995). The cingulate cortex receives inputs from the medial and lateral thalamic nuclei, which contain nociceptive neurons. These studies, therefore, indicate a specific role of the anterior cingulate cortex in pain processing which is a particular event and probably related to other functions of this brain region such as attention and learning. In a recent meta-analysis of studies using brain imaging techniques, Jensen et al. (2016) also demonstrated a common and central role of the anterior cingulate cortex in processing different types of pain (Jensen et al., 2016).

The formalin-induced bilateral activations observed in our study are consistent with findings from other studies using both P.E.T. and fMRI (Tuor et al., 2000; Malisza et al., 2003; Shih et al., 2008). Shih et al. (2008) observed that formalin-induced nociception leads to increased activation in the cingulate cortex, somatosensory cortex, thalamus, and amygdala (Shih et al., 2008). Casey, (1999) showed that subcutaneous injection of formalin leads to selective and progressive activation of the somatosensory and limbic systems, including the insula, anterior cingulate cortex, thalamus, premotor cortex and cerebellum, all of these areas in a bilateral way (Casey, 1999). The absence of lateralisation in the cerebral activation observed in our study and other authors’ data suggests that integration through the two cerebral hemispheres occurs in the central processing of the pain. However, the main afferent pathways reach the upper portions of the brain by contralateral routes. Bilateral activation of these areas is possibly associated with the animal representation of mirror-shaped pain in which contralateral paw licking is also observed after the addition of formalin (Aloisi et al., 1993).

The Ph*α*1*β* toxin has been shown to possess analgesic action in several preclinical pain models in rodents (Diniz et al., 2014). Its potent analgesic action is associated with the inhibition of voltage-sensitive calcium channels (Vieira et al., 2005) and inhibition of TRPA1 receptors (Tonello et al., 2017) present in presynaptic nerve terminals leading to a reduction of glutamate release in the spinal cord (Souza et al., 2008) and also inhibiting the activation of afferent sensory neurons (Castro-Junior et al., 2013; Tonello et al., 2017). Our study is the first to demonstrate the brain activation profile involved with the analgesic action of Ph*α*1*β*. Notably, Ph*α*1*β* attenuated formalin-induced brain activation in the cingulate cortex and motor cortex. In both regions, the inhibition occurred predominantly in the hemisphere contralateral to the pain stimulus. Presumably, the inhibition of neurotransmission caused by Phα1β in the spinal synapses (Vieira et al., 2003; Vieira et al., 2005) is associated with a decrease in primary afferent inputs to be processed in the motor and sensory cortex, which receive information from circuits that are contralateral to the nociceptive stimulus. Lin et al. (2014) observed that gabapentin, another calcium channel blocker, was able to reverse metabolic changes in the prefrontal cortex, thalamus, and cerebellum, induced by sciatic nerve injury in rats (Lin et al., 2014). Gabapentin also acts as a blocker of voltage-sensitive calcium channels, and the difference between the areas inhibited by Phα1β and gabapentin in the different studies can be explained by the pain model used in both studies (acute x neuropathic), and also by the administration of drugs (intrathecal for Ph*α*1*β* and systemic for gabapentin). Also, Ph*α*1*β* acts predominantly on N-subtype channels and is more closely related to nociceptive stimuli’ afferent sensory transmission. Gabapentin, by binding on α2δ subunit of calcium channels, may have caused broader inhibition of neurotransmission in the C.N.S.

We evaluated the effect of Morphine on formalin-induced metabolic changes in the brain in comparison with Ph*α*1*β*. Like Ph*α*1*β*, Morphine attenuated the activation of the cingulate cortex and motor cortex more expressively in the contralateral hemisphere (although not statistically significant). However, Morphine significantly inhibited the amygdala in comparison to Phα1β. These data on the effect of Morphine in the amygdala are consistent with the data of Robincohen et al. (1991) which showed that Morphine decreases the of glucose uptake in regions of the limbic system and therefore morphine is able to reverse methabolic activation of those regions (Robincohen et al., 1991). The amygdala is a bilateral structure located deep and medially within the temporal lobes of the brain. It plays a crucial role in the processes of memory, decision-making and emotional reactions, being considered part of the limbic system (Amunts et al., 2005). The lack of effect of Phα1β on the amygdala suggests that the analgesic action of this toxin may be associated with a lower emotional depressant effect when compared to Morphine. Shih et al. (2008), in a similar experimental design, observed that the pretreatment with Morphine was able to inhibit brain activation in all areas analysed, with inhibition of the order of 40%, while the inhibition of Morphine in our study were close to 10%. These differences can be explained by the morphine dosage difference employed or by the experimental design. Shi et al. applied the dose of 10 mg/kg intravenously. In our study, we used the 10 μg/site of Morphine by intrathecal route. Also, we induced nociceptive process with formalin after the injection of ^18^F-FDG, whereas Shih et al. (2008) administered formalin before ^18^F-FDG injection. When analysed in parallel, the two models possibly lead to different uptake profiles induced by the nociceptive process since the formalin-induced pain response has a short duration. Ohashi et al. (2007) also observed a reversal of metabolic activation in the brain caused by Morphine in a visceral hypersensitivity model. However, in the latter study, more pronounced morphine effects were observed in the thalamus and sensory cortex (Ohashi et al., 2007).

Our study includes some limitations. ^18^F-FDG P.E.T. cannot discriminate inhibitory from excitatory brain activity since both activities consume energy and increase glucose’s metabolic consumption. The deactivation of a given neural network may reflect the reduced neuronal activity. However, it is not possible to differentiate whether this deactivation is caused by the suppression of glutamatergic cells or gabaergic cells’ activation. Secondly, our acquisitions of P. E. T. images are static and, therefore, do not contain information on the dynamics of ^18^F-FDG capture. Finally, the process of capturing images is done under anaesthesia with inhaled isoflurane. Although this stage is crucial to allow immobilisation of the animal during the pictures, it is possible that this anaesthetic action may underestimate the extent of the brain activation induced by the pain process.

## Conclusion

In conclusion, this study presents a brain map of formalin-induced nociception, revealing that several regions are activated and possibly involved in central pain processing. Also, the analgesic power of the Ph*α*1*β* peptide and Morphine is associated with the attenuation of metabolic activation in different brain areas. This study contributes to the preclinical development of analgesic drugs and helps elucidate a common area of pain processing.

## Data Availability

The original contributions presented in the study are included in the article/supplementary materials, further inquiries can be directed to the corresponding author.

## Abbreviations

18F-FDG, fluorodeoxyglucose; CB, cerebellum; FMRI, functional magnetic resonance imaging; Gm, gray matter; Pag, periaqueductal gray; PET, positron emission tomography; Suv, Standardized Uptake Value; Voi, volumes of interest; Vta, ventral tegmental area; Wm, white matter.
